# Associations of mitochondrial DNA 3777–4679 region mutations with maternally inherited essential hypertensive subjects in China

**DOI:** 10.1186/s12881-020-01045-7

**Published:** 2020-05-15

**Authors:** Ye Zhu, Jia You, Chao Xu, Xiang Gu

**Affiliations:** 1 Clinical Medical College, Yangzhou University, Yangzhou, 225001 Jiangsu China; 2 Department of Cardiology, Northern Jiangsu People’s Hospital, Nantong West Road No.98, Yangzhou, 225001 Jiangsu China; 3 Department of Internal Medicine, Yangzhou Maternal and Child HealthCare Hospital, Yangzhou, 225001 Jiangsu China; 4 Department of Biostatistics and Epidemiology, University of Oklahoma Health Science Center, Oklahoma City, OK 73104 USA

**Keywords:** Mitochondria, DNA, Mutation, Essential hypertension, Maternal inheritance

## Abstract

**Background:**

Nuclear genome or family mitochondrial screening system has become the hot focus of studies into essential hypertension. The role of mitochondrial DNA (mtDNA) in sporadic Chinese patients with hypertension has not been fully understood. The study was to evaluate the associations of mtDNA mutations with maternally inherited essential hypertensive subjects in China.

**Methods:**

From June 2009 to June 2016, a total of 800 gender-matched Chinese patients with maternally inherited essential hypertension (MIEH) and control group were 1:1 enrolled in this case-control study. Genomic DNA was extracted from each person’s peripheral blood cells. The main mtDNA locations for MIEH were screened with oligodeoxynucleotides 3777-4679 bp, analyzed and compared with the updated consensus Cambridge Sequence. Pathogenic mtDNA mutations were identified from the mitochondrial map.

**Results:**

MIEH subjects presented significantly higher values than those of control group in abdominal circumference (AC), waist circumference (WC), body mass index (BMI), fasting blood glucose (FBG), triglyceride (TG), low-density lipoprotein cholesterol (LDL) and renal function (*P* < 0.05). MIEH subjects carried more amino acid changes and coding sequence variants (*P* < 0.01) than control group. The allele frequencies of the eight single nucleotide polymorphisms (SNPs) were significantly different between the two groups, including *m.3970 C > T, m.4048G > A, m.4071C > T, m.4086C > T, m. 4164A > G and m.4248 T > C* in ND1 gene, and *m.4386 T > C* and *m.4394C > T* in tRNA^Gln^ gene(*P* < 0.001). Fifty-five homoplasmic or heteroplasmic mutations were detected in 5 genes: ND1, tRNA^Ile^, tRNA^Met^, tRNA^Gln^ and ND2 gene. The ND1 gene was the main mutation site, where the most mtDNA mutation was *m.3970 C > T.*

**Conclusions:**

The mtDNA mutations were involved in the process of MIEH. We identified mitochondrial genetic characteristics in MIEH patients in China. The present research serves as a solid foundation for further detailed research on the association between MIEH and mitochondrial dysfunction, and their causal relationship in Chinese and other populations with a similar lifestyle.

## Background

Essential hypertension (EH) is a common cardiovascular disorder, influencing about 1 billion people around the world [1]. It is generally believed that the interaction between genic and environmental factors affected EH, which may be caused by single-gene defects or multifactorial conditions [2]. Maternally inherited essential hypertension (MIEH) is EH that is consistent with the pattern of maternal inheritance [3]. Mitochondrial DNA (mtDNA) can cause mitochondrial diseases, which are transmitted from the mother exclusively. MtDNA mutations were marked in several pathogenic disorders including mitochondrial myopathy, stroke-like attacks, encephalopathy and maternally hereditary diabetes [4]. In addition, mutations in mtDNA have also been observed to play a role in the pathogenesis of MIEH [5].

The repair and protection systems of mtDNA are less efficient compared to that of nuclear DNA [6]. MtDNA mutations have been linked to MIEH through modifying several functional tRNAs [7]. In particular, a previous study identified that the mutation may reduce the steady-state level of mt-tRNA^Gln^*m.4375C > T* and subsequently cause the mitochondrial dysfunction that is responsible for hypertension [8]. So far, the mechanism of mtDNA mutations in MIEH has not been completely elucidated, especially on the interplay between mtDNA mutations and other risk factors, such as the development of blood pressure, nuclear genes, and environmental conditions [9]. While people are more concerned about the role of the nuclear genome [10], investigating the role of mtDNA sequence alteration may help to understand the genetic pathogenesis of MIEH.

Mitochondrial genes in 3777–4679 region were proposed to be hot spots for mutations associated with hypertension as described previously [11]. In order to better understand the pathogenic mechanisms underlying MIEH, we studied clinical and genetic evidence to investigate the association between the mtDNA mutations in 3777–4679 region and MIEH. In this study, we focused on the Han Chinese population, as there is a limited amount of study on this racial group and they might be overlooked for lacking medical knowledge and regular examinations [12].

## Methods

### Subjects

The current case-control study was based on 400 unrelated patients with MIEH and 400 healthy control individuals in the Jiangsu Province of China. The MIEH patients were recruited according to the following inclusion criteria:

1. in-patients or outpatients who have undergone regular medical check-up at the Department of Cardiology in Northern Jiangsu People’s Hospital from June 2009 to June 2016;
2. more than 18 years old;
3. with a diagnosis of primary hypertension;
4. diagnosed with MIEH on the basis of the maternal transmission of EH within generations, which was transmitted by the mother or her relatives, rather than by the father.

Participants were excluded if they were diagnosed as follows:

1. secondary hypertension (e.g. aortic coarctation, renal arterial stenosis, hyperaldosteronism, and pheochromocytoma);
2. congenital cardiovascular disease;
3. organic valve diseases.

Another 400 gender-matched healthy individuals were recruited to the current study as controls. Furthermore, the controls were unrelated healthy subjects from the same area who received annual examination in physical examination center of Northern Jiangsu People’s Hospital. They were collected randomly from the physical examination list. The control group included the following criteria:

1. systolic blood pressure (SBP) of < 130 mmHg and diastolic blood pressure (DBP) of < 85 mmHg.
2. no personal or family history of hypertension.

Hypertension in one or both biologic parents was considered to be a positive family history of EH. All subjects in the study were interviewed to identify both personal and family medical histories of clinical abnormalities. Verbal Informed Consent, medical history, clinical assessment and genetic analysis were obtained from each individual under protocols involved in the study. Verbal consent is that the mtDNA analysis is used only for diagnosis, not for treatment. It was of no harm to anyone. The protocol was implemented in accordance with the Declaration of Helsinki and approved by the ethics committee of the institutional review board at the Northern Jiangsu People’s Hospital.

### Data collection

Body mass index (BMI) refers to a person’s body mass in kilograms divided by height in square meters (kg/m^2^). Patients reporting cigarette use within 1 year prior to examination were considered as smokers. Blood pressure was measured by an experienced physician who was blinded to the study according to the criteria of the World Health Organization (WHO) [13]. Three measurements of systolic and diastolic blood pressure were taken and the mean value was used as the measurement. According to the 2010 Chinese Hypertension Management, hypertension was diagnosed as follows [14]: the SBP > 140 mmHg and/or DBP > 90 mmHg measured three times on different days or a history of hypertension with current antihypertensive medications. All participants also underwent laboratory tests on hypertension risk factors. 12 h after fasting, lipid profile, fasting blood glucose (FBG), and kidney function test were performed by an automatic biochemistry analyzer (Hitach 7600DDP, Japan).

### Mitochondrial DNA analysis

Genomic DNA was extracted from each person’s peripheral blood using standard protocols [15]. MtDNA was isolated by Promega Wizard Genomic DNA Purification Kit (Madison, WI, USA). The main chromosome locations for hypertension as described previously [16] were screened using oligodeoxynucleotides 3777-4679 bp. The mitochondrial tRNA^Ile^ gene was amplified by Polymerase chain reaction (PCR) using the primer sequences as follows: forward: 5′- TGGCTCCTTTAACCTCTCCA-3′ and reverse: 5′- AAGGATTATGGATGCGGTTG -3′. PCR cycle program was carried out in a 9700 Thermocycler (Perkin-Elmer Applied Biosystems, Norwalk, USA). Each fragment had been purified and sequenced by ABI 3730 Sequence Analysis software (Applied Biosystems, Inc., Foster City, CA, USA) using the BigDye Terminator v1.1 kit (ABI Company, Carlsbad, CA, USA), and subsequently SeqWeb program GAP (GCG) was analyzed and compared with the updated consensus Cambridge Sequence [17, 18]. Pathogenic mitochondrial variants were identified from the human mitochondrial genome database (https://www.mitomap.org/MITOMAP) [19].

### Statistical analysis

Statistical analysis was performed using R and SPSS software (version 22.0; SPSS Inc., Chicago, IL, USA). Continuous variables were tested for normal distribution by Kolmogorov-Smirnov test and then expressed by mean ± standard deviation (SD). The relationship between potential continuous and discrete factors and MIEH were analyzed with Student’s t-test and Fisher’s exact t-test. A *P*-value ≤0.05 was considered to be statistically significant.

## Results

Clinical evaluation of baseline characteristics.

Table 1 shows the main characteristics of all participants. In the present study, age and SBP did not differ between the two groups. There were significant differences in BMI, waist circumference (WC), abdominal circumference (AC), total cholesterol (TC), triglycerides (TG), low-density lipoprotein cholesterol (LDL), FBG, uric acid (UA), creatinine (CR) as well as blood urea nitrogen (BUN) (*P* < 0.05) between the two groups.

**Table 1:** Comparison of baseline clinical data between the MIEH and control groups

Subjects	MIEH group	Control group	*P-*value
gender (M/F)	400 (202/198)	400 (197/203)	1
age at test (years)	68.65 ± 8.34	65.36 ± 6.75	1.38e-9
age at onset (years)	48.56 ± 6.7	NA	
SBP (mmHg)	148.5 ± 19.8	145.6 ± 18.6	0.033
DBP (mmHg)	94.8 ± 8.9	88.4 ± 12.5	3.72e-16
BMI (kg/m^2^)	25.90 ± 3.60	23.52 ± 3.03	1.11e-22
WC (cm)	86.90 ± 10.78	78.08 ± 8.71	8.52e-34
AC (cm)	89.50 ± 11.15	80.28 ± 7.79	2.02e-37
Alcohol, n (%)	100(25)	41(10)	5.13e-8
Smoking, n(%)	90(23)	52(13)	5.84e-4
TG (mmol/L)	1.86 ± 1.20	1.37 ± 0.88	8.65e-11
TC (mmol/L)	4.58 ± 1.96	4.25 ± 1.78	0.0129
LDL (mmol/L)	2.63 ± 1.24	2.02 ± 1.39	1.04e-10
FBG (mmol/L)	5.19 ± 2.18	4.33 ± 1.84	2.54e-09
UA (umol/L)	368.04 ± 127.28	323.39 ± 78.92	4.02e-09
Cr (ummol/L)	105.39 ± 33.71	87.38 ± 30.71	9.40e-15
BUN (mmol/L)	5.76 ± 2.08	4.84 ± 1.79	3.86e-11

Abbreviations: *F* female; *M* male. *SBP* Systolic blood pressure; *DBP* Diastolic blood pressure; *BMI* Body mass index; *WC* waist circumference; *AC* abdomen circumference; *TG* triglyceride; *TC* total cholesterol; *LDL* low-density lipoprotein cholesterol; *FBG* fasting blood glucose; *UA* uric acid; *Cr* creatinine; *BUN* blood urea nitrogen; *: A *P* value < 0.05 was marked by a star

### mtDNA analysis

**Fig. 1:**
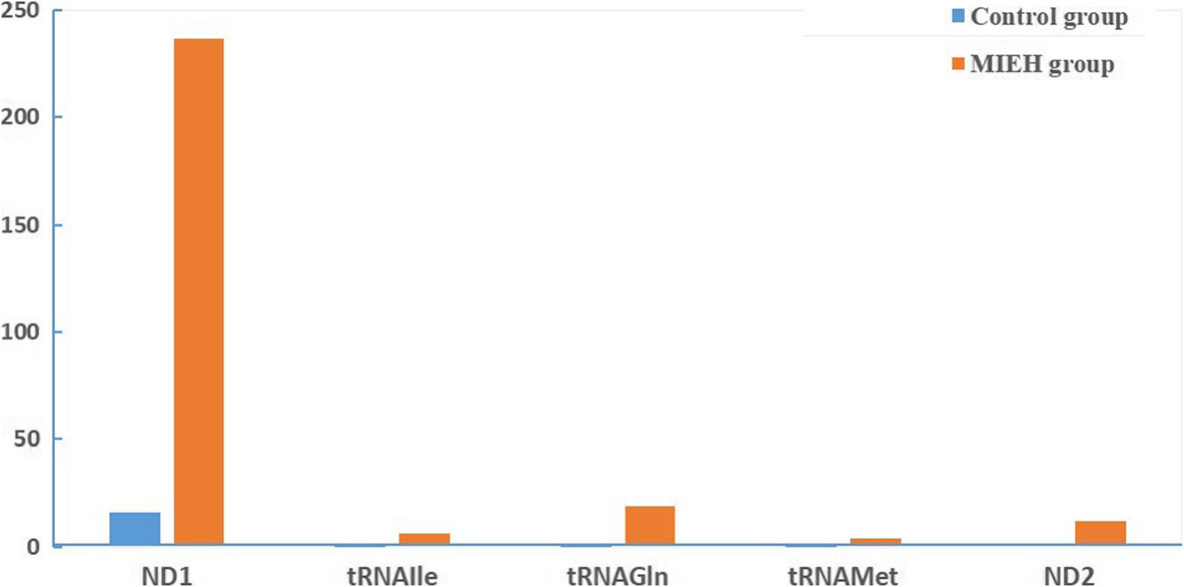
Distribution histogram of the number of variants in mtDNA 3777–4679 bp among 5 genes

**Fig. 2:**
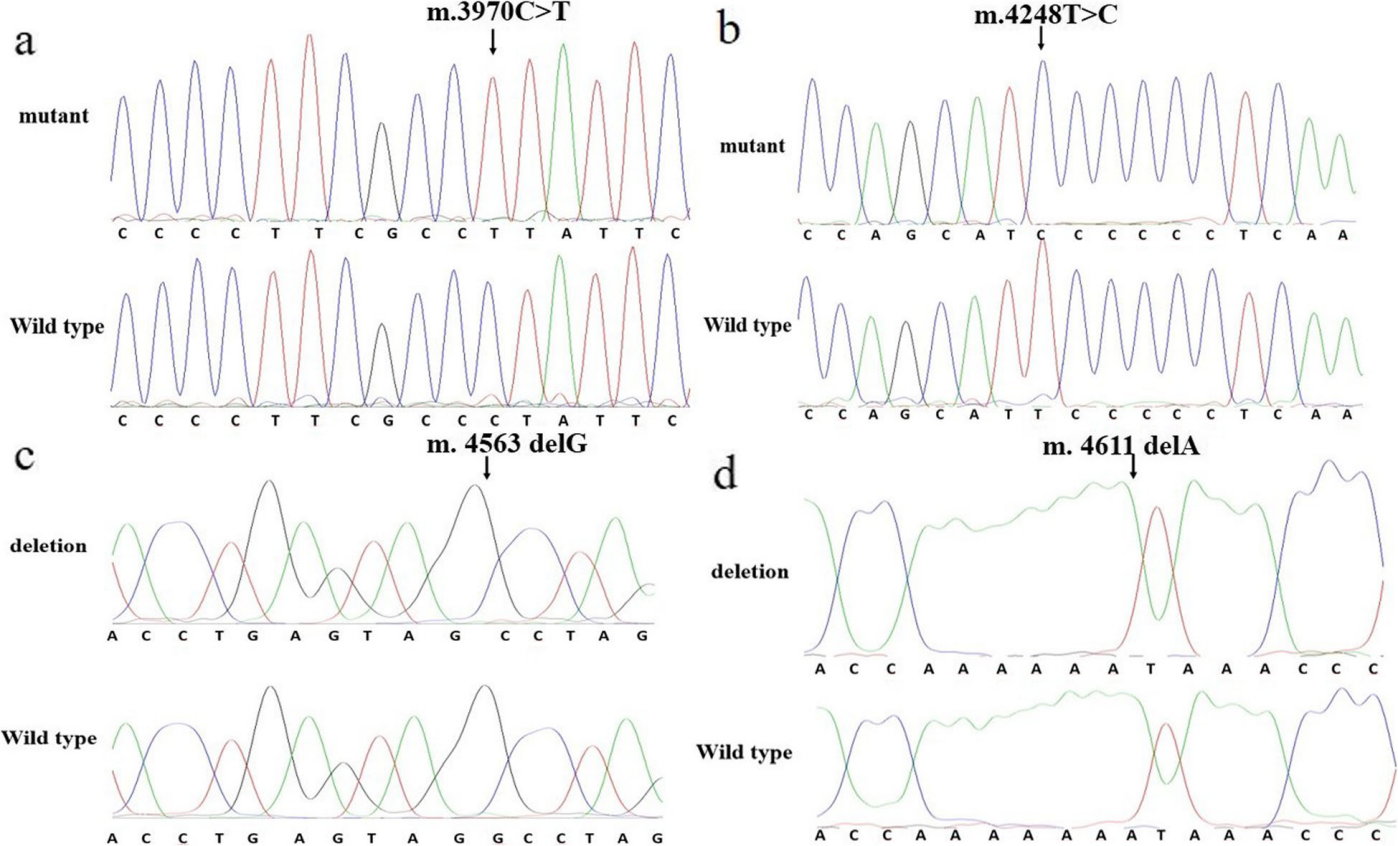
**a**. Identification of the *m.3970C > T* mutation in the mitochondrial ND1 gene. Arrow indicates the position of the gene mutation. **b**. Identification of the *m.4248 T > C* mutation in the mitochondrial ND1 gene. Arrow indicates the position of the gene mutation. **c**. Identification of the *m.4563 delG* mutation. Arrow indicates the position of the deletion mutation. **d**. Identification of the *m.4611delA* mutations. Arrow indicates the position of the deletion mutation

The distribution of mutations in the mtDNA 3777–4679 bp of all participants is shown in Fig. 1. Table 2 shows a comparison of the frequency of mtDNA sequence analyses in the 400 balanced cases and controls. MtDNA analysis revealed 55 mutation sites in the 400 MIEH subjects (Table 3). The ND1 gene was the main mutation site, where the highest mutation frequency was *m.3970 C > T* (Fig. 2a).

**Table 2:** Distribution of mtDNA sequence analyses at positions 3777–4679

Gene	Position	Length	Control group (n(%))	MIEH group (n(%))	Fisher’s exact *P* value
ND1	3777–4262	486	16	237	< 2.2e-16
tRNA^Ile^	4263–4331	69	0	6	0.0307
tRNA^Gln^	4329–4400	72	0	19	3.065e-06
tRNA^Met^	4402–4469	68	0	4	0.1241
ND2	4470–4679	210	1	12	0.003185

**Table 3:** Mutation sites of mtDNA in MIEH individuals and controls

Site of mutation	Gene	Replacement	Homoplasmic/heteroplasmic variants	Number of mutations(n)		P value	Previously reported^a^	Change of Amino acid
				(MIEH)	(Controls)			
3902	ND1	A to T	heteroplasmic	1	0	1	NO	non-syn;D-V
3905	ND1	T to A	heteroplasmic	1	0	1	NO	Non-syn: L-H
3915	ND1	G to A	homoplasmic	6	1	0.1234	YES	Syn: G-G
3918	ND1	A to G	homoplasmic	3	1	0.6241	YES	Syn: E-E
3941	ND1	A to G	homoplasmic	1	0	1	NO	Non-syn: N-S
3948	ND1	A to G	homoplasmic	1	0	1	YES	Syn:E-E
3970	ND1	C to T	homoplasmic	67	5	4.72e-16	YES	Syn:L-L
4017	ND1	C to T	homoplasmic	1	0	1	YES	Syn:L-L
4025	ND1	C to T	homoplasmic	3	1	0.6241	YES	Non-syn: T-M
4038	ND1	A to G	homoplasmic	2	0	0.4994	YES	Syn:G-G
4047	ND1	T to C	homoplasmic	1	0	1	YES	Syn:Y-Y
4048	ND1	G to A	homoplasmic	20	2	9.93e-05	YES	Non-syn: D-N
4071	ND1	C to T	homoplasmic	24	2	7.77e-06	YES	Syn:Y-Y
4083	ND1	T to C	homoplasmic	1	0	1	YES	Syn:F-F
4086	ND1	C to T	homoplasmic	19	1	3.29e-05	YES	Syn:V-V
4092	ND1	G to A	homoplasmic	1	0	1	YES	Syn:K-K
4093	ND1	A to G	homoplasmic	1	0	1	YES	Non-syn: T-A
4104	ND1	A to G	homoplasmic	1	0	1	YES	Syn:L-L
4113	ND1	G to A	homoplasmic	1	0	1	YES	Syn:L-L
4116	ND1	C to T	homoplasmic	1	0	1	YES	Syn:F-F
4117	ND1	T to C	homoplasmic	1	0	1	YES	Syn:L-L
4129	ND1	A to G	homoplasmic	3	0	0.2491	YES	Non-syn: T-A
4131	ND1	A to T	heteroplasmic	1	0	1	NO	Syn:T-T
4134	ND1	A to T	heteroplasmic	1	0	1	NO	syn: A-A
4135	ND1	T to C	homoplasmic	1	0	1	YES	Non-syn: Y-H
4136	ND1	A to G	homoplasmic	1	0	1	YES	Non-syn: Y-H
4140	ND1	C to T	homoplasmic	1	0	1	YES	Syn:P-P
4161	ND1	C to T	homoplasmic	1	0	1	YES	Syn:L-L
4164	ND1	A to G	homoplasmic	14	1	8.83e-4	YES	Syn:M-M
4170	ND1	C to T	homoplasmic	1	0	1	YES	Syn:L-L
4176	ND1	A to G	homoplasmic	2	0	0.4994	YES	Syn:W-W
4200	ND1	A to T	heteroplasmic	2	0	0.4994	YES	Syn:L-L
4203	ND1	A to G	homoplasmic	4	0	0.1241	YES	Syn:A-A
4216	ND1	T to C	homoplasmic	4	0	0.1241	YES	Non-syn: Y-H
4227	ND1	A to G	homoplasmic	1	0	1	YES	syn: M-M
4233	ND1	T to C	homoplasmic	1	0	1	YES	Non-syn: I-T
4245	ND1	C to T	homoplasmic	2	0	0.4994	YES	Syn:S-S
4248	ND1	T to C	homoplasmic	39	2	3.25e-10	YES	Syn:I-I
4254	ND1	T to C	homoplasmic	1	0	1	YES	Syn:P-P
4314	tRNA^Ile^	T to C	homoplasmic	4	0	0.1241	YES	tRNA
4317	tRNA^Ile^	A to G	homoplasmic	2	0	0.4994	YES	tRNA
4336	tRNA^Gln^	T to C	homoplasmic	1	0	1	YES	tRNA
4386	tRNA^Gln^	T to C	homoplasmic	8	0	0.0075	YES	tRNA
4394	tRNA^Gln^	C to T	homoplasmic	10	0	0.0018	YES	tRNA
4401	tRNA^Met^	A to G	homoplasmic	1	0	1	NO	tRNA
4435	tRNA^Met^	A to G	homoplasmic	1	0	1	YES	tRNA
4452	tRNA^Met^	T to C	homoplasmic	1	0	1	YES	tRNA
4457	tRNA^Met^	C to A	heteroplasmic	1	0	1	NO	tRNA
4491	ND2	G to A	homoplasmic	4	1	0.3734	YES	Non-syn: V-I
4515	ND2	G to C	heteroplasmic	1	0	1	NO	Non-syn: G-R
4535	ND2	A to C	heteroplasmic	2	0	0.4994	NO	Syn:L-L
4562	ND2	A to G	homoplasmic	2	0	0.4994	YES	Syn:V-V
4563	ND2	G to T	heteroplasmic	1	0	1	NO	Syn:G-C
4563	ND2	delG		3	0	0.2491	YES	
4574	ND2	A to T	heteroplasmic	1	0	1	NO	Syn:M-M
4576	ND2	delA		1	0	1	YES	
4580	ND2	G to A	homoplasmic	1	0	1	YES	Syn:M-M
4611	ND2	delA		10	0	0.0018	YES	
4612	ND2	delT		2	0	0.4994	YES	

^a^See https://www.mitomap.org/MITOMAP

These results showed that the mtDNA in the MIEH group had more variations than the control group. The allele frequencies of eight single nucleotide polymorphisms (SNPs) were significantly (*P* < 0.001) different between the two groups, including *m.3970 C > T*, *m.4048G > A*, *m.4071C > T*, *m.4086C > T*, *m.4164A > G* and *m.4248 T > C* (Fig. 2b) in ND1 gene, and *m.4386 T > C* and *m.4394C > T* in tRNA^Gln^ gene. Fifty-five heteroplasmic or homoplasmic mutations were detected in 5 genes: ND1, tRNA^Ile^, tRNA^Met^, tRNA^Gln^ and ND2 gene. We found 45 homoplasmic mutations in 267 subjects of MIEH. Ten heteroplasmic mutations were found in 11 MIEH subjects. MIEH subjects carried more amino acid changes and coding sequence variants (*P* < 0.01) compared with normotensive (NT) individuals. An interesting observation in MIEH patients was that we found 4 deletions: *m.4563 delG* (Fig. 2c), *m.4576 delA*, *m.4611 delA* (Fig. 2d) and *m.4612 delT* mutations in MIEH group patients and the site of highest deletion frequency (10/400 = 0.025) was *m.4611 delA* mutation in 10 MIEH subjects. The results suggested that mtDNA mutations were positively correlated with MIEH.

## Discussion

The purpose of this study was to investigate mutations in mtDNA 3777–4679 region of the Chinese MIEH population. The results showed that the group of MIEH had more mtDNA variations, which mainly located at ND1 site and the highest mutation site was *m.3970C > T*. Mitochondrial dysfunction is an important factor in cardiovascular disorders [20]. As far as we know, this is one of the first large-scale Han Chinese population-based studies about and the potential role of mtDNA in MIEH. The systematic screen of the association between MIEH and mtDNA mutation is not only essential to further our understanding of the specific mechanism of the mutation in disease etiology, but can also improve the diagnosis and treatment of hypertension.

In an attempt to determine whether mtDNA was involved in the biochemical indicators of the MIEH individuals, we compared and analyzed the biochemical abnormalities of all the participants. Clinical evaluation of all the participants suggested that MIEH subjects presented significantly higher values than those of control group in BMI, WC, AC, LDL, TG, FBG and renal function. Notably, MIEH individuals were obese or overweight in comparison to NT group. High BMI for EH was reported as a strong predictor of hypertension in a multivariate analysis [21]. Therefore, a normal body weight (BMI 18.5–24.9 kg/m^2^) should be recommended for the prevention and management of MIEH [22]. These factors might contribute to the occurrence and development of MIEH, or they might occur as a result of the progress of MIEH, which results in the damage of the target organ.

Many studies have depicted the function of inherited mtDNA mutations in MIEH family, such as the analysis of maternally transmitted hypertension in a large Han Chinese cohort [23]. Here, we investigated the mtDNA 3777–4679 region using PCR amplification and the sequence analysis. Current results showed that eight SNPs were significantly different between the MIEH and the control groups: *m.3970C > T*, *m.4048G > A*, *m.4071C > T*, *m.4086C > T*, *m.4164A > G* and *m.4248 T > C* in ND1 gene, *m.4386 T > C* and *m.4394C > T* in tRNA^Gln^ gene. ND1 gene is a hotspot for mutations linked to MIEH. Notably, the impaired synthesis of ND1 (subunits of OXPHOS complex I) may be specifically responsible for the decreased activities of complex I [24]. Mitochondrial encoded complex I is a crucial component in the respiratory chain. The ND1 variants in complex I early in the OXPHOS process, which may contribute to the production of reactive oxygen species (ROS) and may participate in key functional development processes of EH [25].

Mutations of mtDNA may lead to disease, and the significant determinant of their clinical manifestation are susceptible to associated with mutations arising in mtDNA [26]. In this study, unlike mild elevation of total cellular ROS production, an increase in the cells carrying the *m.4248 T > C* and *m.3970 C > T* mutations suggested that the defective mitochondria are the major producers of ROS. In turn, the increased levels of cytosolic ROS may produce damage to mitochondrial proteins, nucleic acids, stimulating a forward feeding loop of mitochondrial ROS generation and aggravated cell damage [27]. The reduced levels of mitochondrial proteins were observed in cell lines carrying hypertension associated homoplasmic tRNA^Gln^*m.4386 T > C* and *m. 4394 C > T* mutations belonging to mitochondrial haplogroup M. Ancestral variations in human mtDNA define population-specific mtDNA lineages or haplogroups. Importantly, these were used to trace the origins of different races and provide a foundation for mitochondria based evolutionary medicine [28]. The association between mtDNA haplogroup/SNPs and hypertension has been attributed to a significant alteration in the structure of tRNA and may decrease the steady-state levels of tRNA and oxygen consumption rates that may affect protein levels [29]. Failing to keep the balance of oxygen consumption and production induces mitochondrial dysfunction which was implicated in hypertension pathophysiology, as mentioned earlier [30]. Therefore, mtDNA mutations are promising novel biomarkers for the early detection, prevention, and management of MIEH.

MtDNA mutations, incorporating point mutations, deletions that affect transcription and translation of mtDNA are implicated in various mitochondrial disorders. Rocha et al. have previously revealed a strong correlation between mitochondrial genetics and respiratory chain deficiency in patients with single, large-scale mtDNA deletion [31]. In line with the previous report, our intriguing observation is that there were also a lot of sporadic single mtDNA deletions in MIEH patients [32]. The mean onset time of hypertension for patients with mtDNA point mutations and deletions was gradually ahead of schedule. Thus, it suggests that mtDNA is probably the molecular cause of this disorder. Deletions arise in the mtDNA of post-mitotic cells in patients with mtDNA maintenance disorders, especially during aging. The impaired mitochondrial function may contribute to increased blood pressure of aging with the accumulated multiple mtDNA deletions later in life. The later onset of clinical symptoms due to mtDNA deletions is likely due to the multiple copies of mtDNA within cells. Therefore, the time for mtDNA deletions exceeded an acritical biochemical threshold in proportion, which is necessary to cause a bioenergetic deficit. The mtDNA deletions are thought to be caused by copying errors or repair, or a combination of the both, with the rate of mtDNA deletion formation likely depends on the disorder, the tissue and mechanism of mtDNA replication [32]. We found that the mtDNA deletions disrupted most of the MT-ND2 genes. This suggests there may be duplications with breakpoints that affect complex I, explaining their isolated complex I defect, or there are other deletions we have not found yet. A possible explanation of mtDNA defects in hypertension is that clonally expanded mtDNA deletions are known to be an underlying cause of mitochondrial OXPHOS deficiency in post-mitotic cells. There are studies demonstrating higher levels of mtDNA deletion heteroplasmy in cytochrome c oxidase deficient cells, ultimately initiating hypertension [33]. Some specific deletions in mitochondria DNA may have an underlying role in Chinese hypertensives due to their dysfunction. How mitochondrial mutations and/or deletions interact synergistically in the phenotypic manifestation of hypertension is the next question, which needs further investigation. However, there is still uncertainty regarding the exact mechanisms by which mtDNA deletions clonally expand in patients with mtDNA maintenance disorders. The present study was the primitive step in evaluating the role of mitochondria in unrelated Chinese hypertension patients.

We observed some limitations in this study. Our result from the single-center with relatively small sample size needs further validation. We studied the mtDNA from peripheral blood, which may not represent the mitochondrial activity in other tissues [34]. Another limitation was the lack of functional experiments. Further investigations should be performed to determine the mitochondrial dysfunctions caused by mtDNA mutations, including assessment of ROS production, ATP production and so on.

## Conclusion

The mtDNA mutations were involved in the pathological process of MIEH. Additionally, we identified the mitochondrial genetic characteristics of MIEH patients in Chinese Han population. The investigation of the role of mitochondrial dysfunction in MIEH provides new insights into the understanding and treatment of the disorder. The present research serves as a solid foundation for further detailed research on the association between MIEH and mitochondrial dysfunction, and their causal relationship in Chinese and other populations with a similar lifestyle.

## Data Availability

The raw data of mitochondrial map reported in this paper have been deposited in the Genome Sequence Archive (Genomics, Proteomics & Bioinformatics 2017) in BIG Data Center (Nucleic Acids Res 2019), Beijing Institute of Genomics (BIG), Chinese Academy of Sciences, under accession numbers CRA002605 that are publicly accessible at https://bigd.big.ac.cn/gsa. The updated consensus Cambridge Sequence and pathogenic mitochondrial variants were identified from the human mitochondrial genome database (https://www.mitomap.org/MITOMAP).

## Abbreviations

MtDNA: Mitochondrial DNA
MIEH: Maternally inherited essential hypertension
EH: Essential hypertension
SBP: Systolic blood pressure
DBP: Diastolic blood pressure
BMI: Body mass index
WC: Waist circumference
AC: Abdominal circumference
